# Increased alpha-synuclein tear fluid levels in patients with Parkinson’s disease

**DOI:** 10.1038/s41598-020-65503-1

**Published:** 2020-05-22

**Authors:** Fabian Maass, Sebastian Rikker, Vivian Dambeck, Carmina Warth, Lars Tatenhorst, Ilona Csoti, Matthias Schmitz, Inga Zerr, Andreas Leha, Mathias Bähr, Paul Lingor

**Affiliations:** 1 Department of Neurology, University Medical Center, Göttingen, Germany; 2 Gertrudis Clinic Parkinson-Center, Biskirchen, Germany; 3 DZNE, German Center for Neurodegenerative Diseases, Munich and Göttingen, Germany; 4 Department of Medical Statistics, University Medical Center, Göttingen, Germany; 5 Cluster of Excellence Nanoscale Microscopy and Molecular Physiology of the Brain (CNMPB), Göttingen, Germany; 6 Center for Biostructural Imaging of Neurodegeneration (BIN), University Medical Center, Göttingen, Germany; 7 Technical University of Munich, School of Medicine, Klinikum rechts der Isar, Department of Neurology, 81675 Munich, Germany

**Keywords:** Parkinson's disease, Diagnostic markers

## Abstract

The objective of the study was to estimate if altered levels of alpha-synuclein can be detected in tear fluid of patients with Parkinson’s disease (PD). Therefore, tear fluid samples of 75 PD patients, 75 control subjects and 31 atypical Parkinsonian patients were collected and analyzed in triplicates using an ultra-sensitive single molecule array (SIMOA) system and applying a human alpha-synuclein immunoassay. In PD, levels of total soluble alpha-synuclein were significantly increased compared to control subjects (p = 0.03; AUC PD vs. controls 0.60). There was no difference comparing PD patients stratified by Hoehn & Yahr stages and atypical Parkinsonian syndromes stratified by tauopathies and non-PD-synucleinopathies against each other (p > 0.05). In conclusion, alpha-synuclein can be detected and quantified in tear fluid, revealing small but significant differences in total alpha-synuclein levels between PD and control subjects. Tear fluid can be collected non-invasively and risk-free, therefore presenting a promising source for further biomarker research.

## Introduction

Alpha-synuclein (aSyn) is an essential component of the Lewy body and represents the hallmark protein of Parkinson’s disease (PD) pathology^1^. Consequently, the potential of aSyn to function as a biomarker for PD was analyzed in multiple different biofluids (e.g. serum and CSF). However, high intra- and interstudy variability in aSyn levels and the lack of discriminatory power prevent its use as individual biomarker for diagnosis of PD^2^.

Previously, aSyn positivity in PD was demonstrated in the salivary glands^3^, but saliva itself did not yield a clear difference in aSyn levels between PD and controls^4^, also due to preanalytical difficulties (e.g. blood contamination, bioactive enzymes^5^).

**Figure 1:**
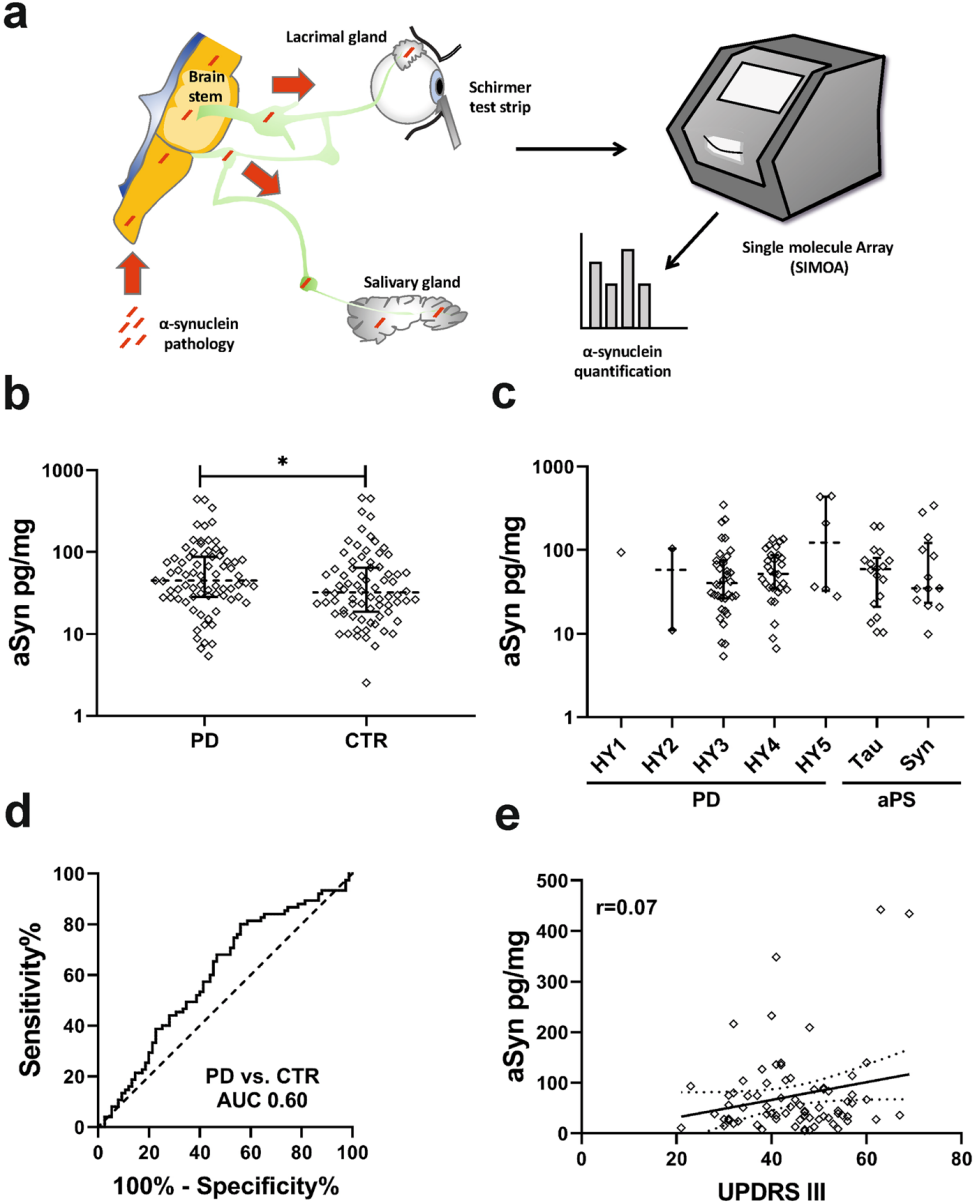
(**a**) The lacrimal gland and the salivary gland share the same parasympathetic innervation originating in the brainstem yielding a common connection for the transmission of aSyn pathology. Tear fluid was collected using Schirmer strips. aSyn levels were quantified applying single molecule array (SIMOA) (**b**) aSyn tear fluid levels in PD/CTR (p = 0.03). (**c**) aSyn levels in PD stratified by Hoehn & Yahr stage (HY) and in atypical Parkinsonian (aPS) syndromes stratified by tauopathies (Tau) and synucleinopathies (Syn). (**d**) ROC curve for the discrimination of PD and CTR. (**e**) Correlation between aSyn and UPDRS III. Data in (**b**,**c**) is presented as single values with median and interquartile range on a log10 scaled y-axis. PD = Parkinson’s disease, CTR = control subjects.

The lacrimal gland and the salivary gland share the same parasympathetic innervation originating in the brainstem yielding a common connection for the transmission of aSyn pathology (Fig. 1a). Tear fluid (TF) is a cost efficient, easily and non-invasively collectable body fluid. Its analysis thus presents a promising alternative approach considering its superior preanalytical characteristics.

Recently, a multiplex ELISA was used to quantify aSyn levels in TF^6^. However, conventional ELISA-based analyses have yielded inconsistent results on aSyn biofluid levels in the past (e.g. conflicting aSyn plasma levels^7^) whereas the application of ultrasensitive single molecule array (SIMOA) might yield more sensitive and reliable quantification results, as recently described for the detection of aSyn plasma levels in PD^8^.

Here we present the first study of aSyn quantification in TF using SIMOA in a comprehensive cohort of PD patients, control subjects and atypical Parkinsonian syndromes.

## Results

150 participants (PD n = 75, CTR n = 75) were enrolled for primary analysis. There was no significant difference in gender, age or ophthalmological comorbidities between PD and CTR (p > 0.05, summarized in Table 1). Additionally, 18 patients with tauopathies (PSP n = 13, CBS n = 5) and 13 patients with other synucleinopathies (MSA-C n = 2, MSA-P n = 11) were included for secondary analysis.

**Table 1:** Characteristics of the study population.

	PD	CTR	P-value^#^	Tauo-pathies*	Non-PD Synucleino-pathies^§^
Patients (*n)*	75	75		18	13
Age *(years)*	70 (64–76)	70 (62–79)	0.77	71 (67–77)	71 (66–78)
Male/female *(% female)*	49/26 (34.7%)	50/25 (33.3%)	0.86	10/8 (44.4%)	8/5 (38.5%)
H&Y stage	3.0 (3.0–4.0)	NA		4.0 (3.4–4.0)	3.0 (3.0–4.0)
Disease duration *(years)*	7.0 (4.0–10.0)	NA		2.5 (2.0–4.0)	4.0 (2.0–6.0)
UPDRS III	45 (38–52)	NA		39 (31–52)	36 (27–54)
Glaucoma *n (%)*	1 (1.3%)	4 (5.3%)	0.17	0 (0.0%)	0 (0.0%)
Macular degeneration (*n) (%)*	1 (1.3%)	1 (1.3%)	1.00	1 (5.6%)	0 (0.0%)
Cataract (*n) (%)*	9 (12.0%)	7 (9.3%)	0.60	1 (5.6%)	0 (0.0%)
Any other eye disease *(n) (%)*	6 (8.0%)	7 (9.3%)	0.77	1 (5.6%)	1 (7.7%)
Contact lenses *n (%)*	3 (4.0%)	1 (1.3%)	0.31	0 (0.0%)	0 (0.0%)
Medical eye drops *n (%)*	5 (6.7%)	6 (8.0%)	0.75	2 (11.1%)	0 (0.0%)
Lubricants *(n) (%)*	10 (13.3%)	4 (5.3%)	0.09	2 (11.1%)	0 (0.0%)

Data is presented as median (25^th^– 75^th^ percentile). PD = Parkinson’s disease, CTR = control subjects, NA = not applicable. UPDRS = Unified Parkinson’s Disease Rating Scale.

^**#**^P-values reported for the comparison of PD and CTR.

*Tauopathies including PSP n = 13, CBS n = 5.

^**§**^Synucleinopathies including MSA-C n = 2, MSA-P n = 11.

There was neither a significant difference in TF total protein concentrations (2.10 µg/µl, IQR 1.65–2.86 vs. 2.07 µg/µl, IQR 1.31–2.75; p = 0.64) nor in sum wetting length (15 mm, IQR 8–24 vs. 15 mm, IQR 8–28; p = 0.55) comparing PD and CTR.

In PD, levels of aSyn were significantly increased compared to CTR (44.91 pg/mg, IQR 28.26–87.16 vs. 32.02 pg/mg, IQR 18.57–64.51; p = 0.03; mean triplicate CV 7.9%; Fig. 1b), resulting in a moderate discriminative value with an AUC of 0.60 (p = 0.03, 95% CI 0.51–0.69, sensitivity 0.44/specificity 0.80 on Youden index; Fig. 1d).

There was no difference in aSyn levels comparing PD patients stratified by H&Y stages and atypical Parkinsonian syndromes stratified by tauopathies and non-PD-synucleinopathies against each other (H&Y1 93.54 pg/mg, IQR NA; H&Y2 57.58 pg/mg, IQR 11.09–104.1; H&Y3 40.21 pg/mg, IQR 26.35–75.19; H&Y4 51.86 pg/mg, IQR 34.63–86.89; H&Y5 122.8 pg/mg, IQR 32.55–436.3; tauopathies 59.12 pg/mg, IQR 20.83–80.35; synucleinopathies 34.78 pg/mg, IQR 23.31–121.2; p = 0.73; Fig. 1c).

There was a weak correlation between age and aSyn levels in CTR (r = 0.28; p = 0.02) but not in PD (r = 0.19; p = 0.10). aSyn levels were not significantly correlated with disease duration or UPDRS III score (r = 0.05, p = 0.70; r = 0.07, p = 0.57, Fig. 1e).

A binary logistic regression model was fitted to distinguish PD from CTR based on aSyn levels and potentially confounding factors age, gender, application of medical eye drops or lubricants, presence of eye diseases or usage of contact lenses. This model still yielded a significant difference in aSyn between PD and CTR (p = 0.048) without significant confounding effects by the mentioned factors.

## Discussion

In a proof-of-principle approach, we demonstrate that aSyn can be detected and quantified in TF by ultra-sensitive SIMOA, revealing increased values of total soluble aSyn in PD compared to control subjects. Quantification was performed in multiple measurements (triplicates) per subject, yielding a mean triplicate CV of 7.9%.

The performance of SIMOA for aSyn detection in tear fluid should be validated in further trials and currently, there is no gold standard available which could be referred to. Previous studies reporting aSyn concentrations in other biofluids used different in-house developed or commercially available ELISA systems^2,9^. Here, we applied SIMOA instead of conventional ELISA due to its known capability for ultrasensitive protein measurements and increasing evidence for superior detection characteristics in protein quantification, e.g. for the quantification of neurofilaments^10,11^.

For the current study, we preferred the application of Schirmer strips for collection and analysis of reflex tears instead of the usage of microcapillary tubes or anesthesia for the collection of basal tears. Using Schirmer strips is common in clinical practice and has many advantages compared to capillary tube sampling^12^. It is very safe and well tolerated by the patients, therefore it can be easily translated into clinical routine. Avoiding anesthesia additionally allows to exclude bias due to potential influences by the medication.

The source of TF aSyn is uncertain and a release into TF by innervating nerve structures in the course of aSyn transmission might be involved. Being an ultrafiltrate of plasma^13^, TF aSyn might also be blood derived. Ng and colleagues recently applied SIMOA for the detection of total soluble aSyn in plasma samples showing results similar to our TF data, revealing slightly increased aSyn values in PD and a distinct overlap with the control group, respectively^8^.

Only a few studies have analyzed TF in PD and recently aSyn was shown to be decreased in TF of PD patients^6^. In this study, however, anesthetic eye drops were instilled prior to performance of the Schirmer test and aSyn levels were quantified using an ELISA instead of an ultra-sensitive SIMOA.

We further report that aSyn can be detected in TF of patients with atypical Parkinsonian syndromes. Our analysis of an exploratory cohort showed no clear difference in aSyn values between tauopathies (PSP, CBD), non-PD-synucleinopathies (MSA-P, MSA-C) and PD. Here, a larger number of these less frequently occurring patients needs to be analyzed in order to draw robust conclusions. Nevertheless, we demonstrate that the measurements in both groups of atypical Parkinsonian patients are in a similar range compared to PD patients.

We detected no significant differences in aSyn values in PD patients stratified for H&Y stages, which is comparable to the results that were reported for aSyn plasma levels quantified by SIMOA^8^. However, most patients were classified H&Y 3 or 4, and we thus cannot exclude that patients in other stages could show more pronounced changes. Likewise, there was also no significant correlation between aSyn levels and UPDRS III motor score.

With its moderate discriminative value (AUC 0.60), total soluble TF aSyn will not be sufficient as an individual biomarker for diagnosis, but the value of easily collectable TF remains promising. Further validation in independent and larger cohorts including patients with other disease entities is needed to confirm our results.

So far, only one further group recently reported total aSyn quantification in tear fluid, applying a Luminex ELISA in contrast to our SIMOA approach^6^. Both approaches allow for alpha-synuclein detection, yielding evidence for the occurrence of aSyn in TF accompanied by small differences in total aSyn levels between PD and control subjects (AUC 0.63 and AUC 0.60, respectively). Distinct preanalytical and analytical differences prevent direct comparison of both studies (application of anesthetic eye drops, different types of antibodies, different detection and quantificationmethods). Taking together the data from both studies, it can be assumed that total aSyn will not be sufficient as a diagnostic biomarker for the discrimination of PD patients and controls. However, aSyn could be part of a biomarker panel and its reliable detection in an easily accessible biofluid, such as tear fluid, is therefore of particular interest^14^.

As we could recently show, TF can also be analyzed by proteomics and PD patients show a differential protein composition as compared to controls^15^. Therefore, protein combinations could potentially act as biomarkers rather than individual proteins. In addition, the analysis of aggregated aSyn isoforms using protein-misfolding cyclic amplification (PMCA) or real-time quaking-induced conversion (RT-QuIC)^16^ could complement SIMOA to improve discriminative power for the translation of an individual biomarker into clinical routine.

## Methods

### Participants

PD patients diagnosed according to MDS criteria^17^ and control subjects (CTR) without clinical signs of neurodegeneration but comparable age- and gender characteristics were consecutively enrolled at the out- and in-patient clinics of the Department of Neurology of the University Medical Center Göttingen and at the Gertrudis Parkinson Center Biskirchen, Germany between August 2018 and May 2019 for primary analysis. All patients were included independent of disease severity. Patients with Progressive Supranuclear Palsy (PSP), Corticobasal Syndrome (CBS) and Multiple System Atrophy (MSA-P, MSA-C) according to acknowledged criteria^18–20^ were also enrolled in this period and additionally selected from the biobank of the Department of Neurology, Göttingen based on availability of TF samples. Patients underwent neurological examination and history taking by movement disorder specialists. Ophthalmological comorbidities and topical eye medication were recorded. UPDRS score part III was applied for assessment of motor function.

### Ethical approval and informed consent

A permission of the local ethics committee has been obtained prior to the initiation of the study (Ethics Committee of the University Medical Center Göttingen Nr.: 13/11/12; 10/8/18). All participants voluntarily participated in the study. Informed consent for study participation was obtained from all patients and control subjects. The study conforms with the Code of Ethics of the World Medical Association (Declaration of Helsinki).

### Sample collection and preparation

TF samples were collected from both eyes using Schirmer test strips (Optitech, Allahabad, India). Strips were placed at the inferior eye lid margin for 8 min and wetting length was noted, respectively. No topical anesthesia was used for sample collection. Samples were immediately frozen after collection and stored in polypropylene tubes at −80 °C until further analysis.

TF protein was eluted by adding RIPA buffer (Thermo Fisher Scientific, Waltham, MA) with protease and phosphatase inhibitor (cOmplete and PhosStOP; Roche, Basel, Switzerland) with subsequent centrifugation at 16000 g for 30 min. Samples of both sides were pooled for analysis. Total protein content was determined using a bicinchoninic acid assay (Thermo Fisher Scientific).

Total soluble aSyn was quantified using an ultra-sensitive single molecule array detection system (SR-X, Quanterix, Billerica, MA) applying a human aSyn immunoassay (Discovery Kit 102233, Quanterix; details are given in the supplement). A positive aSyn control (1/3 concentration of manufacturer’s LOQ or lowest calibrator) was additionally added as duplicate on each plate to confirm that potential values between default LOQ (4.12 pg/ml, defined by the manufacturer applying a coefficient of variability (CV) < 20% with 80–120% recovery, mean recovery 101.1%) and 1/3 of default LOQ (1.373 pg/ml; calculated mean intra-assay CV 13.8%, mean inter-assay CV 19.6%, recovery 68–124%, mean recovery 99%) are still sufficient for quantification and further statistical analysis. All measurements were performed in triplicates using a 1:10 dilution. Results were normalized to total TF protein concentrations.

### Statistical analysis

Quantitative data were compared using Mann–Whitney test. Qualitative data were compared using Chi-Squared test. 95% confidence interval (CI) of the area under the ROC curve (AUC) was calculated according Wilson/Brown method. One-way ANOVA on ranks was applied to compare PD stratified by Hoehn and Yahr (H&Y) and atypical Parkinsonian patients. A binary logistic regression model was fitted to log10-transformed data to control for potential confounders. Correlation between two variables was quantified using spearman’s rho. Due to the lack of pre-existing data on TF aSyn quantification at the time of study initiation, valid sample size predetermination was not applicable. All analyses were performed using GraphPad Prism 8.0.1. or the R-language 3.5.3 with corresponding packages.

## Supplementary information

Supplementary Information.

## Data Availability

The datasets analyzed during the current study are available from the corresponding author on reasonable request.
