# Supplementary material for: Increased alpha-synuclein tear fluid levels in patients with Parkinson’s disease
Source: Sci Rep. 2020 May 22;10:8507. doi: 10.1038/s41598-020-65503-1 (PMC7244583; doi:10.1038/s41598-020-65503-1)
Supplement: Supplementary file 1 — Supplementary Information. [file 41598_2020_65503_MOESM1_ESM.pdf]

Increased alpha-synuclein tear fluid levels in patients with Parkinson's disease

(Supplement)

Fabian Maass, MD[1], Sebastian Rikker[1], Vivian Dambeck[1,5,6], Carmina Warth[1,6], Lars Tatenhorst, PhD[1,3,5,6], Ilona Csoti, MD[2], Matthias Schmitz[1,3], PhD, Inga Zerr, MD[1,3], Andreas Leha, PhD[4], Mathias Bähr, MD[1,5], Paul Lingor, MD[1,3,5,6,7]

[1] Department of Neurology, University Medical Center Göttingen, Germany

[2] Gertrudis Clinic Parkinson-Center, Biskirchen, Germany

[3] DZNE, German Center for Neurodegenerative Diseases Göttingen, Germany

[4] Department of Medical Statistics, University Medical Center, Göttingen, Germany

[5] Cluster of Excellence Nanoscale Microscopy and Molecular Physiology of the Brain (CNMPB), Göttingen, Germany

[6] Center for Biostructural Imaging of Neurodegeneration (BIN), University Medical Center Göttingen, Germany

[7] Department of Neurology, Rechts der Isar Hospital of the Technical University Munich, 80333 Munich, Germany

Corresponding author:

Dr. med. Fabian Maass

Department of Neurology, University Medical Center Göttingen,

Robert-Koch-Str. 40, DE-37075 Göttingen, Germany

Phone: + 049 551 39 8398

Email: fabian.maass@med.uni-goettingen.de

General characteristics of Simoa human a-synuclein assay

(as reported by the manufacturer)

**Calibration range**	0-1,000 pg/mL
**Dynamic range**	0-10,000 pg/mL
**Lower limit of detection**	0.955 pg/mL
**Lower limit of quantification**	4.12 pg/mL
**Spike-recovery (serum/plasma)**	86.0%
**Spike-recovery (CSF)**	105.7%
**Dilution Linearity (serum)**	94.7%
**Dilution Linearity (CSF)**	88.0%
**Typical sample volume**	19 µl
**Minimum sample volume**	2.1 µl

Precision characteristics of Simoa Human α-Synuclein immunoassay

(as reported by the manufacturer)

Sample	Mean αSynuclein (pg/mL)	Within Run %CV (n=5)	Between Run %CV (n=5)
Serum panel 1	1933.4	3.1	3.2
Serum panel 2	3325.0	5.2	2.2
Serum panel 3	563	3.1	2.3
